# Molecular Evidence for Metabolically Active Bacteria in the Atmosphere

**DOI:** 10.3389/fmicb.2016.00772

**Published:** 2016-05-24

**Authors:** Ann M. Klein, Brendan J. M. Bohannan, Daniel A. Jaffe, David A. Levin, Jessica L. Green

**Affiliations:** ^1^Institute of Ecology and Evolution, Department of Biology, University of Oregon, Eugene, ORUSA; ^2^Department of Atmospheric Sciences, University of Washington Bothell, Bothell, WAUSA; ^3^Department of Mathematics, University of Oregon, Eugene, ORUSA; ^4^Santa Fe Institute, Santa Fe, NMUSA

**Keywords:** atmosphere, metabolic activity, rarity, sampling theory, Rhodospirillales

## Abstract

Bacterial metabolisms are responsible for critical chemical transformations in nearly all environments, including oceans, freshwater, and soil. Despite the ubiquity of bacteria in the atmosphere, little is known about the metabolic functioning of atmospheric bacterial communities. To gain a better understanding of the metabolism of bacterial communities in the atmosphere, we used a combined empirical and model-based approach to investigate the structure and composition of potentially active bacterial communities in air sampled at a high elevation research station. We found that the composition of the putatively active bacterial community (assayed via rRNA) differed significantly from the total bacterial community (assayed via rDNA). Rare taxa in the total (rDNA) community were disproportionately active relative to abundant taxa, and members of the order Rhodospirillales had the highest potential for activity. We developed theory to explore the effects of random sampling from the rRNA and rDNA communities on observed differences between the communities. We found that random sampling, particularly in cases where active taxa are rare in the rDNA community, will give rise to observed differences in community composition including the occurrence of “phantom taxa”, taxa which are detected in the rRNA community but not the rDNA community. We show that the use of comparative rRNA/rDNA techniques can reveal the structure and composition of the metabolically active portion of bacterial communities. Our observations suggest that metabolically active bacteria exist in the atmosphere and that these communities may be involved in the cycling of organic compounds in the atmosphere.

## Introduction

Studies of microorganisms in terrestrial, aquatic, and host-associated environments have demonstrated that surveying metabolic activity is key to characterizing the functions of microbial communities (e.g., del Giorgio and Scarborough, 1995; Giorgio et al., 1997; Haglund et al., 2003; Lillis et al., 2009; Peris-Bondia et al., 2011; Baldrian et al., 2012). Yet, little is known about the metabolic activity of microbial communities in the atmosphere, an environment that is intimately connected to all biomes spanning the globe. This lack of knowledge is due to both conceptual and technical limitations. Conceptually, the atmosphere has historically been regarded as a dispersal vector for dead, dormant, or inactive cells instead of as a habitat with actively reproducing microbial life (Womack et al., 2010). Technically, low densities of cells have made it difficult to study the activity of airborne microorganisms *in situ* (Behzad et al., 2015). But there is evidence to suggest that airborne bacteria may be metabolically active. Culture-based analyses of bacteria isolated from clouds have shown that bacteria can transform atmospheric compounds including carbon, nitrogen, and oxidative species (Amato et al., 2007a; Hill et al., 2007; Vaïtilingom et al., 2011, 2013). Research has also demonstrated that aerosolized cultured bacterial cells increase their ribosome production, and thus their protein synthesis potential, when supplied carbon substrates in the lab (Krumins et al., 2014). However, due to biases associated with our inability to cultivate most bacterial taxa, culture-based methods do not provide a comprehensive assessment of bacterial diversity in natural environments. The next step in understanding the metabolic functioning of atmospheric bacteria is to apply culture-independent methods to atmospheric samples, targeting active cells, in order to learn about their potential functions.

The vast majority of culture-independent microbiology research relies on sequence analysis of rDNA (i.e., rRNA genes), which provides information regarding the total community of cells (including active, dead, and dormant individuals). In contrast, sequence analysis of rRNA in ribosomes (Schippers and Neretin, 2006; Lennon and Jones, 2011; Blazewicz et al., 2013; Womack et al., 2015) provides information regarding the potentially metabolically active cells in a community because ribosomes are more abundant in active than in dormant cells (Fegatella et al., 1998; Kerkhof and Kemp, 1999). Studies that have combined both rDNA and rRNA data have led to a wide range of ecological insights including how microbial communities respond to environmental change (Barnard et al., 2013), which taxa contribute to key biogeochemical processes (Schostag et al., 2015), and what mechanisms shape microbial community assembly (Zhang et al., 2014). An emergent theme from comparative rDNA and rRNA analyses is that the active and total community can be fundamentally different from one another, in both structure and composition. For example, in many environmental systems it is the rare members of the total community that are dominant in the active community (Jones and Lennon, 2010; Campbell et al., 2011; Baldrian et al., 2012; Hugoni et al., 2013; Wilhelm et al., 2014; Zhang et al., 2014). This suggests that using rDNA data alone may lead to an underestimation of the functional importance of rare taxa (Jones and Lennon, 2010; Campbell et al., 2011; Aanderud et al., 2015).

Despite the utility of comparative rRNA/rDNA studies, there are limitations (see Blazewicz et al., 2013) associated with this technique that must be carefully considered in the analysis and interpretation of results. One technical complication encountered in several studies is the occurrence of “phantom taxa”, here defined as taxa (operational taxonomic units, aka OTUs) that are only observed in the rRNA sequences but not in the rDNA sequences. This is unexpected because the active community is, by definition, a subset of the total community. The occurrence of phantom taxa has been attributed to differences in sample processing in the laboratory (e.g., cDNA synthesis for rRNA but not rDNA; van Gurp et al., 2013) resulting in the introduction of erroneous base pair changes to rRNA samples but not rDNA samples (Lanzén et al., 2011; Mikkonen et al., 2014; Zhen et al., 2015). This could theoretically lead to a situation where an rDNA gene and its’ rRNA would not be similar enough to be grouped in the same OTU. Another potential explanation for the presence of phantom taxa is the combination of insufficient sampling of the total community (as suggested by Kamke et al., 2010; Gaidos et al., 2011) and variation in the level of metabolic activity among taxa. The effects of insufficient sampling and differential activity may be magnified by two factors. First, taxa that are rare in the rDNA community have been observed to be disproportionately active relative to abundant members (Campbell et al., 2011; Hugoni et al., 2013; Hunt et al., 2013). Second, it is more difficult to detect rare taxa in the total (rDNA) community compared to the active community because metabolically active cells may contain 100s–1000s of ribosomes (Bremer and Dennis, 1996; Fegatella et al., 1998) but only 1–15 rDNA gene copies (Klappenbach et al., 2001). These factors – in isolation or in combination – could contribute to the observation of phantom taxa.

In this study, we applied comparative 16S rDNA and rRNA sequence analyses to characterize the structure and composition of the active and total bacterial communities in the atmosphere. We then used a model-based approach to explore how sampling affects differences in the composition of rRNA and rDNA communities and to assess the likelihood that the presence of phantom taxa is an artifact of sampling. We focused our study on bacteria because they are abundant in the atmosphere [concentration range from 10^4^ to 10^5^ cells/m^3^ (Burrows et al., 2009)] and, due to their small size, can have atmospheric residence times that are long enough for growth and reproduction to occur (Womack et al., 2010). Our study site is located at a high elevation research station on the summit of Mt. Bachelor, Oregon. Mt. Bachelor is an ideal site for studying the potential activity of airborne microbial communities. Due to the geography of the mountain and the surrounding topography, the summit (elevation 2.8 km above sea level) regularly encounters air masses from the free troposphere, making it possible to collect cells which have been aloft in the atmosphere for over 1 week (Weiss-Penzias et al., 2006). By collecting cells that have been in the atmosphere for extended periods of time, the active community should more closely reflect the activity of cells in the atmosphere and not activity in potential local source environments such as water or soil. To gain a better understanding of the potential functions of bacterial communities in the atmosphere, we asked the following questions: What is the diversity and composition of rRNA and rDNA airborne bacterial communities, and how do they compare? Is metabolic activity correlated with taxa abundance? Does sampling theory explain differences in the composition of rRNA and rDNA communities?

## Materials and Methods

### Sample Collection

Sampling was conducted over four days (August 13–16, 2013) at the Mt. Bachelor Observatory (MBO; 43.98°N, 121.7°W), a mountaintop research station, 30 km WSW of Bend, OR. MBO is located at the summit of Mt. Bachelor, an inactive volcano, at 2763 m above sea level. At Mt. Bachelor there is a persistent upslope/downslope diurnal flow in summer. This results in exposure to mainly boundary layer from 10:00 am to 8:00 pm and free tropospheric air from 10:00 pm to 8:00 am (McClure et al., 2016). Aerosol samples were collected using SKC Biosamplers (BioSampler SKC Inc.). Inpingers were filled with 20 mL of a water-based preservation solution (LifeGuard Soil Preservation Solution, MO BIO Laboratories, Inc.) to prevent DNase and RNase activity and maintain cells in stasis to allow accurate profiling of the rRNA and rDNA communities. Twenty-four impingers were operated in parallel at 12.5 L/min from approximately 8:00 am – 4:00 pm each day. At the end of each day, the sampling liquid from all impingers was pooled and stored at –20°C yielding one sample per day for a total of four samples. Impingers were rinsed with sterile water, wrapped in aluminum foil, sealed with autoclave tape, and sterilized using an electric pressure cooker.

### Nucleic Acid Isolation and cDNA Synthesis

Samples were transported on dry ice to the University of Oregon where the liquid was thawed and filtered through 0.22 μm cellulose nitrate filters (Nalgene Analytical Test Filter Funnels, Thermo Fisher Scientific). To control for possible contamination of lab reagents, blank samples were generated by filtering unused, unopened LifeGuard Solution through new, sterile filters. Blanks were processed identically to samples including nucleic acid extraction, cDNA synthesis, barcoding, and sequencing. RNA and DNA were co-extracted from filters using the MO BIO PowerWater RNA Isolation Kit according to the manufacturer’s instructions, with the following modifications (MO BIO Laboratories, Inc.). The initial DNase step was omitted. RNA and DNA were eluted in 100 μl elution buffer (QIAGEN) and then divided in half. One ~50 μl aliquot was treated with 1 μl DNase (DNase I, RNase-free, Thermo Fischer Scientific, Inc.) and the other was treated with 2 μl RNase (RNase A, DNase and protease-free, Thermo Fischer Scientific, Inc.). Both reactions were incubated at 37° for 30 min. Reactions were cleaned using the Qiagen MinElute Enzymatic Reaction Cleanup Kit (QIAGEN). DNA was eluted in 100 μl elution buffer and RNA was eluted in 50 μl elution buffer.

cDNA was synthesized from the total RNA extract from samples and blanks using the SuperScript II First-Strand Synthesis System (Invitrogen, Life Technologies Corporation) with random hexamers. All RNA was converted into cDNA in seven synthesis reactions and one reverse transcriptase negative control. The seven cDNA reactions for each sample were pooled, cleaned using Qiagen MinElute Enzymatic Reaction Cleanup Kit (QIAGEN), and eluted in 50 μl elution buffer. To verify all contaminating DNA was destroyed in the DNase reaction, one control reaction without reverse transcriptase was included for each sample. These control reactions failed to amplify by PCR indicating contaminating DNA was destroyed.

### Library Preparation and Sequencing

16S genes (rDNA) and transcripts (rRNA which was converted to cDNA) were amplified using bacterial/archaeal primers 515 forward (5′-GTGTGCCAGCMGCCGCGGTAA-3′) and 806 reverse (5′-GGACTACHVGGGTWTCTAAT-3′) (Caporaso et al., 2012). The reverse primer also contained a 12 base-pair barcode at the 5′ end in order to assign sequences to samples. PCR reactions were performed in triplicate on each DNA and cDNA sample. Each 25 μl reaction contained 0.25 μl Phusion Hot Start II DNA polymerase (Life Technologies Corporation) 5 μl HF buffer, 0.5 μl 10 mM dNTPs (New England Biolabs, Inc.) 0.5 μl forward primer (10 μM), and 0.5 μl reverse primer (10 μM), 13.25 μl H_2_O, and 5 μl template. PCR cycling conditions were as follows: initial denaturation at 98°C for 90 s followed by 35 cycles at 98°C for 20 s, 52°C for 30 s and 72°C for 30 s following by a final extension at 72°C for 10 min. Triplicate reactions were pooled, cleaned using the MinElute 96 UF PCR Purification Kit (QIAGEN), and eluted in 20 μl elution buffer. The cleaned PCR products were quantitated using a Qubit 2.0 Fluorometer (Invitrogen, Life Technologies Corporation) and combined in equal molar concentrations. The pooled library was concentrated (Zymo Research Clean and Concentrate-5, Zymo Research) and eluted into 50 μl elution buffer. PCR products were size fractionated by gel electrophoresis (2%, low-melt agarose). Products in the range of 250–350 bp were excised, and DNA from the excised gel was extracted (Qiagen MinElute Gel Extraction, QIAGEN) and eluted into 30 μl elution buffer. The eluate was cleaned a final time (Zymo Research Clean and Concentrate-5, Zymo Research) and eluted into 30 μl elution buffer. The final library was quantitated and diluted from 59.42 to 10 nM. The amplicon library was sequenced (300 base pairs, single-end) on the Illumina MiSeq platform at the Dana-Farber Cancer Institute Molecular Biology Core (Boston, MA, USA).

### Sequence Processing

Sequences were processed using QIIME version 1.8 (Caporaso et al., 2010) and the UPARSE pipeline (USEARCH version 7.0.1090) (Edgar, 2013). Briefly, libraries were demultiplexed in QIIME using the split_libraries_fastq.py without quality filtering or trimming. Sequences in fastq output from QIIME were quality filtered and trimmed using the fastq_filter USEARCH script. Sequences were trimmed to 296 bp. Sequences with a maximum expected error (fastq_maxee) >0.5 were removed. Sequences that only occurred once (i.e., singletons) were removed using the derep_fulllength and sortbysize USEARCH scripts. Sequences were clustered into OTUs at 97% sequence similarity using the UCLUST algorithm (Edgar, 2010). Representative sequences from each OTU were screened for chimeras using the uchime_ref script against the ChimeraSlayer reference database (Broad Microbiome Utilities^1^ version microbiomeutil-r20110519). Headers for representative sequences were reformatted and sequences were numbered sequentially using the fasta_number.py script. Sequences were mapped to OTUs using the usearch_global script. The resulting OTU map was converted to a tab-delimited OTU table using a modified version of the uc2otutab.py script. The tab-delimited OTU table was converted to BIOM (McDonald et al., 2012) format. Taxonomy was assigned to representative sequences using the RDP Naïve Bayesian Classifier (Wang et al., 2007) against the Greengenes database^2^ (version 13_5) in QIIME.

OTUs for which relative abundance in control samples was significantly correlated with their relative abundance in air samples were removed using the filter_otus_from_otu_table.py script in QIIME. Of the 90 OTUs that were removed, 26 could not be taxonomically classified. Additionally, all OTUs identified as belonging to the class “Chloroplast” (2,612 sequences, 16 OTUs) or family “Mitochondria” (1,021 sequences, 9 OTUs) were removed using the filter_taxa_from_otu_table.py script.

### Statistical Analyses and Data Availability

All statistical analyses were conducted in R (R Core Team, 2016) primarily using the packages vegan (Oksanen et al., 2016) and BiodiversityR (Kindt and Coe, 2005) for ecological statistics, and the ggplot2 package (Wickham, 2009) for visualizations. All analyses of beta-diversity were based on Canberra distances. DESeq2 (Love et al., 2014) implemented in QIIME was used to analyze differential abundance of OTUs between the rRNA and rDNA communities. Sequence files and metadata have been deposited in Figshare^3^.

### Sampling Model

**FIGURE 1:**
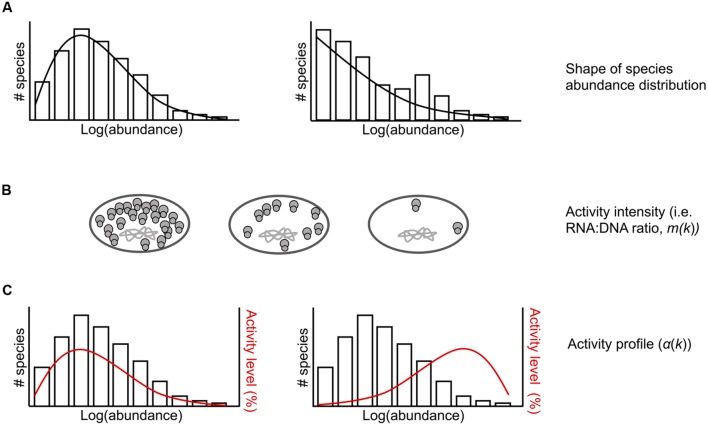
**Conceptual representation of model parameters for the ecological community that is randomly sampled. (A)** Example taxa abundance distributions. The shape of the underlying rDNA taxa abundance distribution can be varied. **(B)** Bacterial cells with chromosomal DNA and varying number of ribosomes per cell. Activity intensity (*m*(*k*)), or the number of rRNAs per rDNA, can be constant or vary across rDNA abundance *k*. **(C)** Example taxa abundance distributions with activity profiles (α(*k*)) shown in red. The activity profile specifies the proportion of active taxa at each abundance *k*. This can be varied such that low abundance values contain a higher proportion of active taxa than high abundant values (left) or vice versa (right).

Sampling models have been widely used in ecology as a tool to understand diversity patterns in communities (Gotelli and Colwell, 2001). Building on this body of literature, we developed sampling theory regarding the joint distribution of rDNA and rRNA in order to gain insights into the effects of sampling on observations of rDNA and rRNA taxa. In brief (details are provided in the Data Sheet S1), we specified the joint distribution by first identifying the taxa-abundance distribution for rDNA, and second, the conditional abundance of rRNA for a given rDNA abundance. For positive integer *k*, the abundance *k* refers to those taxa having *k* individuals in the population. Introducing additional assumptions allows us to identify the conditional abundance of rRNA by providing only the “activity profile” function α(k), the fraction of active (rRNA producing) taxa in (rDNA) with abundance k, and the “activity intensity” function m(k), the ratio of rRNA/rDNA for active taxa with abundance *k* (**Figure 1**). Thus, the model requires as input a taxa-abundance distribution for rDNA, and the functions α(k)and m(k). The qualitative joint behavior of rDNA and rRNA richness in a random sample of individuals from the community, and in particular the frequency of sample “phantom” taxa (those whose rRNA is represented in the sample but whose rDNA is not), is manifested in the rRNA and rDNA rarefaction curves (plots of the number of taxa as a function of the number of individuals sampled) and the curve relating phantom frequency to sampling effort.

## Results

### Diversity and Composition of rRNA and rDNA Communities

**FIGURE 2:**
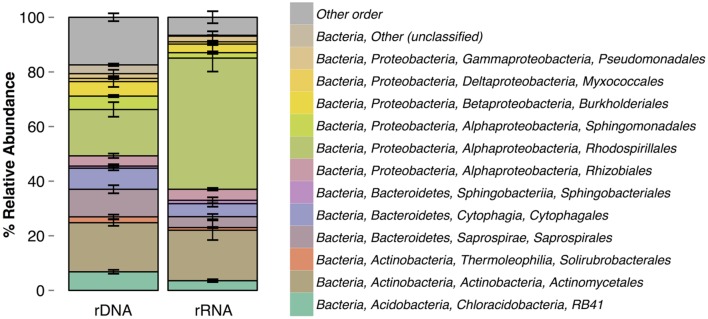
**Order-level taxonomic composition of rRNA and rDNA communities.** Errors bars are standard deviations.

**FIGURE 3:**
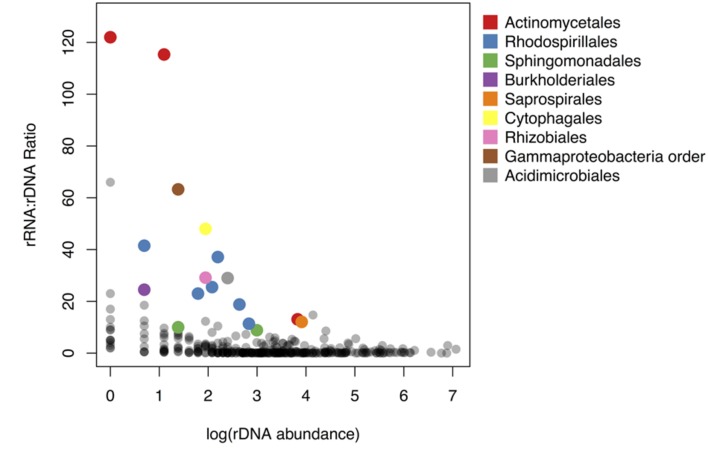
**Relationship between rRNA: rDNA ratio and abundance in the rDNA community.** rRNA: rDNA ratio is analogous to the activity intensity parameter (*m(k)*) in the sampling model. Colored points represent taxa significantly overrepresented in the active community. Points are colored by taxonomic order.

Composition of the rRNA and rDNA communities significantly differed [ADONIS, *F*(1,6) = 1.22, *p* = 0.027] at the level of OTUs and at the order level (**Figure 2**). The rRNA and rDNA communities were dominated by the orders Actinomycetales, RB41, Saprospirales, Cytophagales, and Rhodospirillales. The relative abundances of the following orders significantly differed between rRNA and rDNA: RB41 [*t*(3) = 4, *p* = 0.04], Saprospirales [*t*(3) = 6, *p* = 0.01], Rhodospirillales [*t*(3) = –7, *p* = 0.006], and Sphingomonadales [*t*(3) = 10, *p* = 0.002]. rRNA communities were significantly less variable (as measured by the percentage of shared OTUs) across days than were rDNA communities (*p* < 0.01). rRNA communities shared 28.4 ± 7.2% of OTUs (16.1% of the total cDNA sequences) across all four samples, where as rDNA communities shared an average of 17.6 ± 3.2% of OTUs. Across all OTUs, relative abundance in the rRNA community was correlated with relative abundance in the rDNA community (**Figure 3**, Kendall rank correlation coefficient, tau = –0.078, *p* = 5e-04). The structure (richness, diversity, and evenness) of the rRNA and rDNA communities also differed. Shannon diversity [*t*(3) = 10, *p* = 0.002] and evenness were significantly greater in the rDNA community [*t*(3) = 10, *p* = 0.002]. Richness did not significantly differ [*t*(3) = –0.5, *p* = 0.7, **Table 1**].

**Table 1:** Sequence summary statistics.

Category	Count
Raw sequences	594,783
Filtered sequences	153,088
Mean filtered sequences/sample	19,136 ± 7,708.60
OTUs	1,144
OTUs after rarefaction	1,076
Mean OTUs/library	293.88 ± 39.21
OTUs shared across all libraries	23
OTUs unique to one library	606
Mean unique OTUs/library	75.75 ± 50.26
rDNA OTUs	767
rRNA OTUs	652
OTUs shared between rDNA and rRNA libraries	343 (31.9%)
OTUs in rDNA but not rRNA libraries	424 (39.4%)
OTUs in rRNA but not rDNA libraries (phantoms)	309 (28.7%)

### Taxon Abundance and Activity Potential

Fifty-nine OTUs were identified as differentially abundant (DESeq2, adjusted *p* < 0.05) between the rRNA and rDNA communities, including 12 OTUs that were more abundant in the rDNA community and 47 that were more abundant in the rRNA community. To identify potentially active taxa, we focused our analysis on OTUs that were shared between the rRNA and rDNA communities and that were overrepresented in the rRNA community (DESeq2, *p* < 0.01). These are conservative criteria for activity and may exclude taxa that are only moderately active as well as those that, due to sampling, are not found in both the rDNA and rRNA datasets. Using these criteria, 17 OTUs were identified as potentially metabolically active (Supplementary Table S1).

To compare the potential activity of these 17 OTUs to the remainder of the shared OTUs in the community, we plotted the rRNA: rDNA ratios of all shared OTUs against their abundance in the rDNA community. rRNA: rDNA ratios are frequently used as an index of bacterial activity (e.g., Campbell et al., 2011; Baldrian et al., 2012; Zhang et al., 2014) because the number of ribosome per cell is correlated with growth rate in cultured bacterial isolates (Fegatella et al., 1998; Kerkhof and Kemp, 1999; but see Blazewicz et al., 2013). rRNA: rDNA ratios in this study ranged from 0.002 to 122; approximately 63% of OTUs had rRNA: rDNA ratios less than 1. The taxa with highest rRNA: rDNA ratios were rare members of the rDNA community (**Figure 3**). Specifically, the rRNA: rDNA ratio was negatively correlated with abundance in the rDNA community (Pearson correlation, *r* = –0.11, *p*-value < 0.05).

### Sampling Theory and Phantom Taxa

**FIGURE 4:**
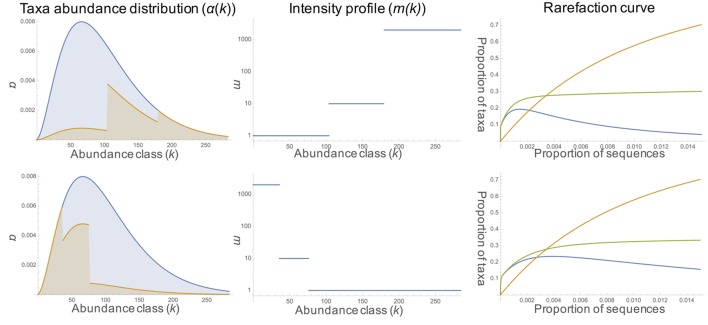
**Model results.** The first column shows the rDNA taxa abundance distribution (negative binomial with shape parameter 1 in blue and the proportion of active taxa in orange [activity profile, α(*k*)]. Column two shows the change in the intensity profile [i.e., rRNA: rDNA ratio, m(*k*)] across abundance classes. Rarefaction curves for rDNA (orange), rRNA (green), and phantoms (blue) are displayed in the third column. In row a, the proportion of active taxa increases (10%, 60%, 100%) with increasing abundance; activity intensity also increases (1, 10, 2000) with increasing abundance class. In row b, the proportion of active taxa decreases with increasing abundance (100%, 60%, 10%); activity intensity also decreases with increasing abundance (2000, 10, 1).

We used three rarefaction curves (rDNA, rRNA, and phantom taxa, **Figure 4**) to describe the expected fraction of total taxa appearing in the sample as a function of sampling rate. The rDNA curve converges to 1 at full sampling intensity; the rRNA rarefaction curve converges to the proportion of total taxa which are active. Under sufficient sampling effort, the number of phantom taxa goes to zero. The rate at which the number of phantom taxa goes to zero depends on the model parameters, including the rDNA abundance distribution, intensity function, and activity profile (**Figure 1**). For example, in communities where the majority of the active taxa are abundant in the rDNA community and activity intensity is positively correlated with rDNA abundance (**Figure 4**, row a), the number of phantoms goes to zero rapidly. In contrast, when active taxa are rare in the rDNA community and activity intensity is negatively correlated with rDNA abundance (**Figure 4**, row b), the number of phantoms does not decrease as rapidly.

## Discussion

In this study we used comparative 16S rDNA and rRNA sequence analyses to ask if there are metabolically active bacteria in the atmosphere. Our results demonstrate that bacteria in the atmosphere contain ribosomes, and some taxa have many more copies of 16S ribosomal RNA than they have 16S ribosomal genes, suggesting that these taxa may be metabolically active. We found that potential activity level (rRNA: rDNA ratio) varies among taxa, resulting in differences in the composition of the rRNA and rDNA communities. Our modeling results support the assumption that differences in the structure and composition of the rRNA and rDNA communities, including the observation of phantom taxa, are due to the sampling of taxa with different levels of activity. Below we explore the composition of rRNA and rDNA communities with a focus on the physiology of potentially active groups, and we conclude with a discussion of the effect of sampling on the observed differences in the rRNA and rDNA communities.

### Rhodospirillales Are Abundant in Both the rRNA and rDNA Communities

Culture-independent tools are making it possible to begin mapping the bacterial composition of the atmosphere on a global scale. While this endeavor remains in its infancy, some general patterns are beginning to emerge. Our rDNA-based bacterial community data is consistent with prior studies and includes several taxonomic orders commonly found in air samples. For example, the Actinomycetales, which were abundant in our samples, are frequently found in the atmosphere (Brodie et al., 2007; Bowers et al., 2013; Gandolfi et al., 2015) and have been found to be abundant in other studies at high elevation sites (Bowers et al., 2012), including MBO (Smith et al., 2013). There are two reasons to expect this order to be abundant in the atmosphere. First, Actinomycetales are abundant and ubiquitous in soil and freshwater (Ventura et al., 2007), and thus there is a large terrestrial source pool. Second, they produce small spores (Reponen et al., 1998), which likely have a long residence time in the atmosphere. We also observed several other taxonomic groups commonly detected in air samples, including Pseudomonadales (Smith et al., 2012), Burkholderiales, and Sphingomonadales (Smith et al., 2013).

One pattern that is unique to our data set is the prevalence of Rhodospirillales. In other studies of airborne bacterial communities, Rhodospirillales have not been found in high relative abundance (Polymenakou et al., 2008; Bowers et al., 2013). One potential mechanism driving the abundance of Rhodospirillales at MBO could be the nearby marine source. MBO is approximately 200 km from the Pacific Ocean, and most air masses travel over the Pacific for several days before reaching the summit (Weiss-Penzias et al., 2006). Atmospheric residence times of marine bacteria collected at MBO may range from approximately 1 day for cells aerosolized at the coast, to several days for cells aerosolized at more distant locations over the Pacific Ocean. Rhodospirillales are frequently found in marine samples (Feingersch et al., 2010; Yin et al., 2013; Li et al., 2014), and their presence in the atmosphere has been reported in a study of communities in the troposphere (~ 8–10 km above sea level; DeLeon-Rodriguez et al., 2013). Researchers found that Rhodospirillales were enriched in samples collected during a hurricane (DeLeon-Rodriguez et al., 2013) suggesting that, under certain conditions, marine bacteria can be aerosolized and reach the upper levels of the atmosphere.

To our knowledge, this is the first rRNA-based analysis of bacteria in the atmosphere. Consistent with what has been reported for other environments, e.g., soil (Baldrian et al., 2012) and water (Wilhelm et al., 2014), we found that the rRNA- and rDNA-community composition differed (**Figure 1**), and our model results demonstrated that observed differences in community composition reflect differences in the metabolic activity among taxa in the atmosphere. Several orders - including RB41 (an uncharacterized order of Acidobacteria), Saprospirales, and Sphingomonadales – were significantly underrepresented in the rRNA relative to the rDNA community and may have reduced activity compared to other taxa. RB41 and Saprospirales are typically found in soils (Janssen, 2006; King et al., 2010), and Saprospirales has also been detected in air samples (Fierer et al., 2008). Sphingomonadales are commonly detected in air samples (Amato et al., 2007b; Bowers et al., 2011; Després et al., 2012) and are abundant on leaf surfaces (Vorholt, 2012). These results suggest that soil and leaf surfaces are likely substantial sources of bacteria in the atmosphere and may shape the composition of the total community, but not necessarily the active community. There was only one order that was significantly more abundant in the rRNA relative to the rDNA community, and this was Rhodospirillales. The potential role of metabolically active Rhodospirillales is discussed below.

### Rhodospirillales Are Potentially Active in the Atmosphere

Of 343 OTUs shared by the rRNA and rDNA datasets (**Table 1**), 59 significantly differed in abundance. Of those, the 14 OTUs that were more abundant in the rRNA than rDNA community belonged to the family Acetobacteraceae. The Acetobacteraceae are members of the order Rhodospirillales. They are known commonly as the acetic acid bacteria, and their metabolism is characterized by the fermentation of ethanol to acetic acid (Komagata et al., 2014). Ethanol is a common chemical in the atmosphere (Kirstine and Galbally, 2012) with emissions from both biogenic (i.e., plant leaves) and anthropogenic sources (Naik et al., 2010) and is a precursor of ozone and peroxyacetyl nitrate, an eye irritant found in smog. This suggests that bacteria in the atmosphere may be involved in the cycling of compounds that are relevant to human health through the biotransformation of ethanol to acetic acid.

The three Acetobacteraceae OTUs with the greatest difference in abundance between the rRNA and rDNA datasets were all identified by BLAST (*E*-value < 1e–100) (Altschul et al., 1990) as *Acidisphaera rubrifaciens. A. rubrifaciens* is an aerobic, chemoorganoheterotroph and facultative phototroph which was originally isolated from an acidic hot spring (Hiraishi et al., 2000). It produces bacteriochlorophyll a and carotenoid pigments, which could protect against UV damage in the atmosphere. Research has shown that optimal growth of *A. rubrifaciens* occurs in the light with simple organic compounds as energy and carbon sources. More specifically, growth can occur on the conjugate bases of organic acids (Hiraishi et al., 2000) found in the atmosphere, such as fumarate, gluconate, lactate, malate, pyruvate, and succinate (Finlayson-Pitts and Pitts, 1999). It is possible that succinate concentrations were elevated during the time of sampling at the MBO site, as there were several active wildfires in the region, and concentrations of succinic acid are often elevated in the atmosphere during biomass burning (Falkovich et al., 2005; Kundu et al., 2010). Furthermore, microbes isolated from the atmosphere have been shown to degrade organic acids, including succinate, and this biological process may be more important than abiotic chemical cycling (i.e., photodegradation; Vaïtilingom et al., 2011). Therefore, the potential ability of airborne bacteria, such as *A. rubrifaciens*, to grow using organic acids has implications for biogeochemical cycling in the atmosphere.

### Rare Taxa in the rDNA Community Were Disproportionately Active

We used rRNA: rDNA ratios to assess the metabolic potential of bacterial taxa in the atmosphere. Ratios ranged from 0.002 to 122 with an average of 3.71 across all taxa detected in both the rRNA and rDNA communities. In contrast, studies in other environments including soil (Dlott et al., 2015) and marine systems (Campbell et al., 2011; Campbell and Kirchman, 2013) have reported lower rRNA: rDNA ratios. For example, rRNA: rDNA ratios in marine systems average have been shown to range from 1.1 to 1.6 (Campbell and Kirchman, 2013), and ratios as high as 10.8 have been reported for a single taxon (Brettar et al., 2012). This suggests that, on average, bacteria in the atmosphere have the same potential for metabolic activity as bacteria in other habitats and may be more active both in terms of the portion of active taxa and the specific activity of individual taxa.

Across all OTUs, abundance in rDNA was correlated with abundance in rRNA. However, OTUs with the highest rRNA: rDNA ratios were all rare members of the rDNA community. In other words, the potential activity of these OTUs was negatively related to abundance in the rDNA community (**Figure 3**). This pattern has been observed in other environments including marine (Campbell et al., 2011; Hugoni et al., 2013; Hunt et al., 2013), freshwater (Wilhelm et al., 2014), and soil systems (Gremion et al., 2003). Across environments, the bacterial taxa which contribute to ecosystem functioning appear rare in rDNA-based surveys, so their importance may not be recognized. This highlights the importance of rRNA-based surveys for linking microbial community composition to ecosystem function, particularly in relatively uncharacterized environments such as the atmosphere.

### Disproportionate Activity of Rare Taxa Drives the Occurrence of Phantom Taxa

Comparative analysis of 16S rRNA and rDNA genes is a useful technique for characterizing the potentially active bacterial community. However, there are a number of caveats which must be considered, such as the fact that ribosome content is not always correlated with growth rate (Blazewicz et al., 2013) and that dormant individuals can contain ribosomes (although generally fewer than active cells; Segev et al., 2012). The interpretation of results using a comparative rRNA/rDNA approach can be further complicated by the observation of phantom taxa. Our model demonstrates that the observation of phantom taxa (which comprised 28.7% of OTUs in this study) is a consequence of sampling stochasticity; rRNA belonging to an active taxon may be included in the sample, while its rDNA avoids collection. Increased sampling of the rDNA community reduces the number of phantom taxa, but the rate at which this occurs depends on the relationship between abundance in the rDNA community and activity level and abundance in the rRNA community. Taxa that are rare in the rDNA community have been shown in a variety of environments to be disproportionately active relative to abundant taxa (Jones and Lennon, 2010; Campbell et al., 2011; Baldrian et al., 2012; Hugoni et al., 2013; Zhang et al., 2014; Wilhelm et al., 2014), and this can contribute to the observation of phantom taxa when using comparative rRNA/rDNA approaches. Based on our model, future comparative rRNA/rDNA studies should consider the expected shape of the rDNA species abundance distribution (SAD), activity intensity and activity profile. Since estimating these metrics is often the goal of rRNA/rDNA sequencing, it may be useful to first obtain these data from a subset of samples. Then, our model could be used to estimate the depth of sampling necessary to reduce the number of phantoms below any prescribed threshold at different activity profiles and intensity functions.

## Conclusion

Airborne metabolically active bacteria may alter the chemistry of the atmosphere through the biogeochemical cycling of organic compounds. However, little is currently known regarding which taxa may be active and their potential functions. Our study represents the first to use both rRNA- and rDNA-based methods to identify potentially active bacteria in the atmosphere. We found that the structure and composition of the rRNA and rDNA communities differed, and the rRNA community was characterized by the presence of a few highly active taxa and many taxa with low activity levels. Taxa that were rare in the rDNA community were the most likely to be metabolically active. Consistent with our empirical data, our model results demonstrated that compositional differences between the rRNA and rDNA communities, including the observation of phantom taxa, could be attributed to random sampling and differences in the activity of taxa and their abundance in the rDNA community.

Using combined rRNA/rDNA sequencing, we were able to identify potentially active taxa in the atmosphere including members of the order Rhodospirillales, specifically *A. rubrifaciens*. *A. rubrifaciens* may be well-suited for growth in the atmosphere because it has pigments that can mitigate UV damage and because it grows well on simple organic compounds commonly found in the atmosphere. We suggest future research should combine both culture-independent and culture-dependent approaches to assess the potential activity of bacteria in the atmosphere. Culture-independent approaches could be used to identify potentially active taxa and then culture-dependent methods could be used to isolate organisms and study their physiology under various conditions (possibly using aerosolization chambers as in Krumins et al., 2014).We also suggest that future studies incorporate sampling through time, particularly over diurnal and seasonal time scales, to gain a better understanding of the relative roles of different environmental variables in structuring the communities. As with any environment, understanding both the structure and function of microbial communities in the atmosphere is needed to assess their potential impact on ecosystem processes such as carbon cycling. This study opens the door for future investigations of the diversity and function of bacterial communities in the atmosphere.

## Author Contributions

AK, BB, DJ, DL, and JG designed the experiments. AK and DL performed the experiments. DL contributed new analytic tools. AK and DL analyzed the data. AK, DL, and JG wrote the manuscript. AK, BB, JL, and DJ, JG reviewed drafts of the manuscript.

## Conflict of Interest Statement

The authors declare that the research was conducted in the absence of any commercial or financial relationships that could be construed as a potential conflict of interest.

## Footnotes

1 http://microbiomeutil.sourceforge.net/

2 http://greengenes.secondgenome.com/downloads/database/13_5

3 https://figshare.com/articles/Sequence_data/2066556
