# Supplementary material for: Molecular Evidence for Metabolically Active Bacteria in the Atmosphere
Source: Front Microbiol. 2016 May 24;7:772. doi: 10.3389/fmicb.2016.00772 (PMC4878314; doi:10.3389/fmicb.2016.00772)
Supplement: Supplementary file 1 [file Table_1.DOCX]

Supplementary Material

Molecular Evidence for Metabolically Active Bacteria in the Atmosphere

**Ann M. Klein^*^, Brendan J. M. Bohannan, Daniel A. Jaffe, David A. Levin, Jessica L. Green**

*** Correspondence:** Corresponding Author: annmaureenklein@gmail.com

# Supplementary Data

**Supplement Table 1.** Fold change, significance, rRNA: rDNA ratios, and taxonomy of OTUs overrepresented in the active community identified using DESeq2.

| log_2_ Fold change | Adjusted p-value | rRNA: rDNA | Taxonomy |
| --- | --- | --- | --- |
| 6.091 | <0.001 | 115.333 | Actinobacteria, Actinobacteria, Actinomycetales, Nocardiaceae |
| 5.259 | <0.001 | 48 | Proteobacteria, Alphaproteobacteria, Rhodospirillales, Acetobacteraceae |
| 4.936 | <0.001 | 63.25 | Actinobacteria, Actinobacteria, Actinomycetales |
| 5.061 | <0.001 | 122 | Proteobacteria, Alphaproteobacteria, Sphingomonadales, Sphingomonadaceae |
| 4.834 | <0.001 | 29 | Actinobacteria, Actinobacteria, Actinomycetales, Microbacteriaceae |
| 4.593 | 0.001 | 41.5 | Proteobacteria, Betaproteobacteria, Burkholderiales, Comamonadaceae |
| 4.394 | 0.001 | 25.5 | Proteobacteria, Alphaproteobacteria, Sphingomonadales, Sphingomonadaceae, Kaistobacter |
| 4.667 | 0.001 | 29.142 | Bacteroidetes, Saprospirae, Saprospirales, Chitinophagaceae |
| 4.823 | 0.001 | 37.111 | Bacteroidetes, Cytophagia, Cytophagales, Cytophagaceae, Hymenobacter |
| 3.721 | 0.002 | 8.85 | Proteobacteria, Alphaproteobacteria, Rhodospirillales, Acetobacteraceae |
| 4.181 | 0.002 | 13.065 | Proteobacteria, Alphaproteobacteria, Rhizobiales |
| 3.950 | 0.003 | 24.5 | Proteobacteria, Alphaproteobacteria, Rhodospirillales, Acetobacteraceae |
| 3.916 | 0.003 | 23 | Proteobacteria, Gammaproteobacteria |
| 3.509 | 0.005 | 10 | Actinobacteria, Acidimicrobiia, Acidimicrobiales |
| 3.878 | 0.005 | 11.353 | Proteobacteria, Alphaproteobacteria, Rhodospirillales, Acetobacteraceae |
| 3.888 | 0.005 | 18.786 | Proteobacteria, Alphaproteobacteria, Rhodospirillales, Acetobacteraceae |
| 3.682 | 0.009 | 12.06 | Proteobacteria, Alphaproteobacteria, Rhodospirillales, Acetobacteraceae, Roseomonas |
