# Supplementary material for: Molecular Evidence for Metabolically Active Bacteria in the Atmosphere
Source: Front Microbiol. 2016 May 24;7:772. doi: 10.3389/fmicb.2016.00772 (PMC4878314; doi:10.3389/fmicb.2016.00772)
Supplement: Supplementary file 2 [file Data_Sheet_1.PDF]

SAMPLING MICROBIAL RDNA/RRNA

1. Model

1.1. Joint rDNA/rRNA distributions. Let $p(k, r)$ be the fraction of OTU's with k rDNA and r (total) rRNA. We can write

$$p(k, r) = p(r \mid k)s(k)\,,$$

where $s(k)$ is the SAD for rDNA and $p(r \mid k)$ is the proportion of OTU's with rDNA-abundance k which have rRNA-abundance r. Define the *activity* function $\alpha(k)$ to be the frequency of active taxa at abundance k. Thus

$$\alpha(k) = 1 - p(0 \mid k)\,.$$

We write

$$p(r) = \sum_{k \geq 0} p(r \mid k)p(k)$$

for the marginal SAD for rRNA. The total fraction of active taxa equals

$$\bar{\alpha} = \sum_{k \geq 0} \alpha(k)s(k) = 1 - p(0)\,.$$

Note that if S is the number of species, then the proportion of total taxa which are active and in abundance class k equals $\alpha(k)s(k)$.

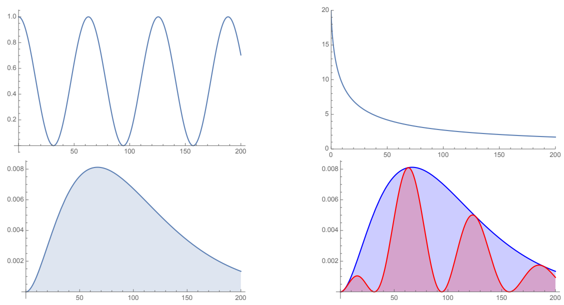

Figure 1. An activity function (top left) α, intensity function m (top right), species abundance distribution (bottom left), and graph of $\alpha(k)s(k)$ (bottom left).

1.2. **Two-point conditional distributions.** For a function $m(k)$, we suppose that

$$p(r \mid k) = (1 - \alpha(k))\delta_0(r) + \alpha(k)\delta_{m(k)k}(r)\,. \tag{1}$$

We call such distribution *simple*. In other words,

$$\begin{aligned} p(0 \mid k) &= 1 - \alpha(k) \\ p(m(k)k \mid k) &= \alpha(k) \\ p(r \mid k) &= 0 \text{ if } r \notin \{0, m(k)k\}\,. \end{aligned}$$

Thus the fraction of active taxa at abundance class k equals $\alpha(k)$, and all active taxa at abundance class k produce exactly $m(k)$ rRNA per individual. We call $m(k)$ the *intensity* function. In Figure 2, all the taxa in red produce $m(k)$ rRNA, while all the blue taxa produce nothing. We can then control how activity varies with abundance by tuning the functions α and m. Note that the total amount of rRNA produced by a single active taxa in abundance class k is $m(k) \cdot k$.

For example, see Figure 2.

2. Generating Functions and Collector Curves

Let

$$\phi_{s,p}(z, w) = \sum_{r,k \geq 0}^{\infty} z^k w^r p(r \mid k) s(k)$$

be the generating function for the joint distribution $\{p(r \mid k)s(k)\}_{r,k\geq 0}$. Then the generating functions for the distributions $\{s(k)\}_{k\geq 0}$ and $\{p(r)\}_{r\geq 0}$ are

$$\phi_s(z) = \sum_{k=0}^{\infty} z^k s(k) = \phi_{s,p}(z, 1), \qquad \phi_p(w) = \sum_{r=0}^{\infty} w^r p(r) = \phi_{s,p}(1, w)\,.$$

If

$$\phi_p(w \mid k) = \sum_{r=0}^{\infty} z^r p(w \mid k)$$

is the conditional probability generating function for the conditional distribution $p(\cdot \mid k)$, then

$$\phi_p(w) = \sum_{r\geq 0} w^r p(r) = \sum_{r=0}^{\infty} w^r \sum_{k=0}^{\infty} p(r \mid k)s(k) = \sum_{k\geq 0} \phi_p(w \mid k)s(k)$$

$$\phi_{s,p}(z, w) = \sum_{k\geq 0} z^k \left[\sum_{r\geq 0} w^r p(r \mid k)\right] s(k) = \sum_{k\geq 0} z^k \phi_p(z \mid k)s(k)\,.$$

Note that $\phi_p(0) = p(0) = 1 - \bar{\alpha}$, the frequency of inactive taxa in the population.

Let us assume that $p(\cdot \mid k)$ is given by the two-point distribution (1). It will be convenient to introduce a third function,

$$\theta(z, w) = \sum_{k \geq 0} (z w^{m(k)})^k \alpha(k) s(k) . \tag{2}$$

We have

$$\phi_p(w \mid k) = (1 - \alpha(k)) + \alpha(k) w^{m(k)k} ,$$

so

$$\begin{aligned} \phi_{s,p}(z, w) &= \sum_{k \geq 0} z^k [1 - \alpha(k) + \alpha(k) w^{m(k)k}] s(k) \\ &= \phi_s(z) - \theta(z, 1) + \theta(z, w) . \end{aligned}$$

It follows that

$$\phi_p(w) = \phi_{s,p}(1, w) = \phi_s(1) - \theta(1, 1) + \theta(1, w) = 1 - \bar{\alpha} + \theta(1, w) .$$

The importance of ϕ_s and ϕ_p come from the collectors' curves for taxa and rRNA, respectively. Let $f_D(\mu)$ and $f_R(\lambda)$ denote the expected proportion of rDNA taxa and rRNA taxa when sampling at rates μ and λ, respectively. Then

$$\begin{aligned} f_D(\mu) &= \sum_{k \geq 0} \left[1 - (1 - \mu)^k \right] s(k) = 1 - \phi_s(1 - \mu) \\ f_R(\lambda) &= 1 - \phi_p(1 - \lambda) \end{aligned}$$

Finally, a taxa appears as a *phantom* if its rRNA appears in the sample but its rDNA does not. The expected proportion of taxa appearing as phantoms at sampling rates μ and λ is

$$\begin{aligned} f_P(\mu, \lambda) &= \sum_{r,k \geq 0} (1 - \mu)^k [1 - (1 - \lambda)^r] p(r \mid k) s(k) \\ &= \phi_s(1 - \mu) - \phi_{s,p}(1 - \mu, 1 - \lambda) \\ &= \theta(1 - \mu, 1) - \theta(1 - \mu, 1 - \lambda) . \end{aligned}$$

It is similarly possible to work out the expected proportion of taxa with DNA only:

$$\begin{aligned} f_{D\star}(\mu, \lambda) &= \sum_{r,k \geq 0} [1 - (1 - \mu)^k] (1 - \lambda)^r p(r \mid k) s(k) \\ &= \phi_p(1 - \lambda) - \phi_{s,p}(1 - \mu, 1 - \lambda) \end{aligned}$$

The expected proportion of shared taxa is given by

$$\begin{aligned} f_{DR}(\mu, \lambda) &= \sum_{r,k} [1 - (1 - \mu)^k][1 - (1 - \lambda)^r] p(r \mid k) s(k) \\ &= 1 - \phi_s(1 - \mu) - \phi_p(1 - \lambda) + \phi_{s,p}(1 - \mu, 1 - \lambda) \end{aligned}$$

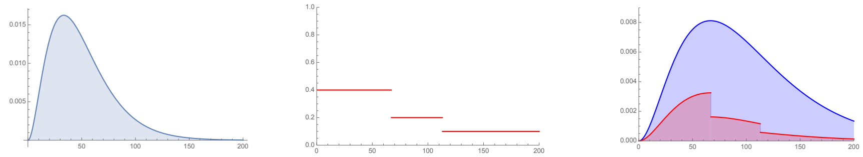

Figure 2. A species abundance distribution (left), a step activity function α (center) , and the corresponding active taxa (right)

$$f_P(\lambda, \mu) = \theta(1-\mu, 1; m) - \theta(1-\mu, 1-\lambda; m)$$

3. Activity and Intensity Step Functions

For arbitrary s, α and m it may be difficult to compute in closed form, or approximate numerically, the generating function $\phi_{s,p}$ and the functions derived from it. For this reason, we consider here simple versions of α and m.

Consider $\beta_0 = 0 < \beta_1 < \cdots \beta_J \leq \beta_{J+1} = 1$ and let k_j be the β_j-th quantile of s. If $\mathbf{1}_I(k) = 1$ if and only if $k \in I$, and zero otherwise, define, for $\{\alpha_i\} \subset [0, 1]$,

$$\alpha(k) = \sum_{j=1}^{J+1} \alpha_j \mathbf{1}_{(k_{j-1}, k_j]}(k) = \begin{cases} \alpha_1 & 0 \leq k \leq k_1 \\ \alpha_2 & k_1 < k \leq k_2 \\ \vdots & \vdots \\ \alpha_{J+1} & k_J < k \leq k_{J+1} . \end{cases}$$

Then

$$\bar{\alpha} = \sum_{k \geq 0} \alpha(k) s(k) = \sum_{j=1}^{J+1} \alpha_j \sum_{k=k_{j-1}+1}^{k_j} s(k) = \sum_{j=0}^{J+1} \alpha_j (\beta_j - \beta_{j-1}) .$$

We also assume m is a step function:

$$m(j) = \sum_{j=1}^{J+1} m_j \mathbf{1}_{(k_j, k_j]}(j) .$$

Define

$$\theta_K(z, w; m_0) = \sum_{k \leq K} (z w^{m_0})^k s(k) .$$

Then

$$\theta(z, w) = \sum_{j=1}^{J+1} \alpha_j [\theta_{k_j}(z, w; m_j) - \theta_{k_{j-1}}(z, w; m_j)]$$

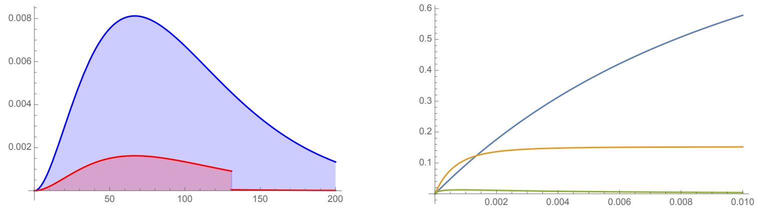

FIGURE 3. Activity profile (left), collector curves (right)

3.1. **Negative Binomial SAD.** We will assume a negative binomial SAD, parameterized by mean η and shape parameter δ. This is a discrete version of the Gamma distribution, and varying η and δ allow for a range of shapes.

The PGF for the distribution is then

$$\phi(z) = \left(1 + \frac{\eta(1-z)}{\delta}\right)^{-\delta} .$$

In this case, we get an expression for $\theta_K(z, w)$ in terms of the Beta function B:

$$\begin{aligned}
\theta_K(z,w) &= \sum_{k \le K} (zw^m)^k \mathrm{NegBinom}(k; \eta, \delta) \\
&= \left(1 + \frac{\eta}{\delta}(1 - zw^m)\right)^{-\delta} \left(1 - (K+1)\binom{K+\delta}{\delta-1} B_{\frac{zw^m\eta}{\delta+\eta}}(K+1, \delta)\right) \\
&= \phi_s(zw^m)\left(1 - (K+1)\binom{K+\delta}{\delta-1} B_{\frac{zw^m\eta}{\delta+\eta}}(K+1,\delta)\right) .
\end{aligned}$$

Thus, if α and m are step functions, we can obtain analytical expressions for all our curves.

4. EXAMPLES

Figure 3 contains a plot of the collector curves (rRNA in gold, phantoms in blue) for the negative binomial $(100, 3)$ distribution, where $\bar{\alpha} = 0.15$ and $\beta = 0.75$. The activity levels are $\alpha_2 = 0.01$ while $\alpha_1 = 0.20$. The average number of rRNA per rDNA is 20. Thus a small fraction of the high abundance (upper quartile) are active, while a higher fraction of the lower abundance (lower 75 percent) taxa are active.

Switching things around so that the lower quartile has low activity (0.01 fraction of taxa are active) and upper three quartiles has high activity (0.20 fraction of such taxa are active) gives the following collector curves; See Figure 4.

It is seen that the behavior of these curves is driven by the different distributional and activity profiles in the population.

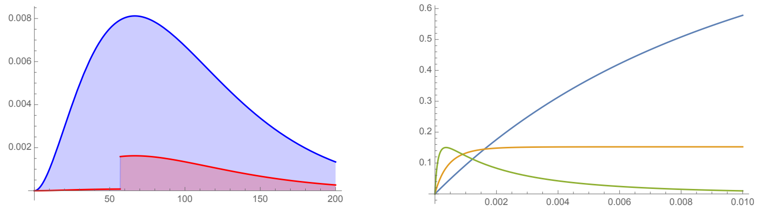

FIGURE 4. Activity profile (left), collector curves (right)
